# Prognostic Value of Comorbidity for Patients with Upper Tract Urothelial Carcinoma after Radical Nephroureterectomy

**DOI:** 10.3390/cancers14061466

**Published:** 2022-03-12

**Authors:** Hung-Lung Ke, Ching-Chia Li, Hsiang-Ying Lee, Hung-Pin Tu, Yu-Ching Wei, Hsin-Chih Yeh, Wen-Jeng Wu, Wei-Ming Li

**Affiliations:** 1 Department of Urology, Kaohsiung Medical University Hospital, Kaohsiung 807, Taiwan; hlke@kmu.edu.tw (H.-L.K.); ccli1010@kmu.edu.tw (C.-C.L.); hsiangying@kmu.edu.tw (H.-Y.L.); patrick@kmu.edu.tw (H.-C.Y.); wejewu@kmu.edu.tw (W.-J.W.); 2 Department of Urology, School of Medicine, College of Medicine, Kaohsiung Medical University, Kaohsiung 807, Taiwan; 3 Graduate Institute of Medicine, College of Medicine, Kaohsiung Medical University, Kaohsiung 807, Taiwan; 4 Department of Urology, Kaohsiung Municipal Ta-Tung Hospital, Kaohsiung 801, Taiwan; 5 Department of Public Health and Environmental Medicine, School of Medicine, College of Medicine, Kaohsiung Medical University, Kaohsiung 807, Taiwan; p915013@kmu.edu.tw; 6 Department of Pathology, School of Medicine, College of Medicine, Kaohsiung Medical University, Kaohsiung 807, Taiwan; ycwei@kmu.edu.tw; 7 Department of Pathology, Kaohsiung Municipal Ta-Tung Hospital, Kaohsiung 801, Taiwan; 8 Center for Liquid Biopsy and Cohort Research, Kaohsiung Medical University, Kaohsiung 807, Taiwan; 9 Department of Urology, Ministry of Health and Welfare, Pingtung Hospital, Pingtung 900, Taiwan

**Keywords:** upper tract urothelial carcinoma, comorbidity, Adult Comorbidity Evaluation-27, prognosis

## Abstract

**Simple Summary:**

Upper tract urothelial carcinoma (UTUC) is a rare malignancy that occurs mostly in elderly individuals with a high prevalence of comorbidities. However, the prognostic impact of comorbidities in these patients is not well evaluated. The aim of this retrospective study was to assess the significance of Adult Comorbidity Evaluation-27 (ACE-27) grade on the clinical outcomes of 409 patients with non-metastatic UTUC who underwent radical nephroureterectomy. We found that a high ACE-27 grade was an independent risk factor for UTUC progression, UTUC-specific death, and all-cause mortality in multivariate analyses. A prognostic model combining ACE-27 grade, tumor stage, and tumor grade showed good predictive performance and accuracy. Integrating the ACE-27 grade with standard pathological features can help physicians in clinical decision-making and risk stratification.

**Abstract:**

Patients with upper tract urothelial carcinoma (UTUC) have a high prevalence of comorbidities. However, the prognostic impact of comorbidities in these patients is not well studied. We aimed to outline the comorbidity burden in UTUC patients and investigate its relationship with overall survival (OS), cancer-specific survival (CSS), and progression-free survival (PFS). We retrospectively reviewed the clinicopathological data of 409 non-metastatic UTUC patients who received radical nephroureterectomy between 2000 and 2015. The comorbidity burden was evaluated using the Adult Comorbidity Evaluation-27 (ACE-27). Kaplan-Meier survival analysis showed that high ACE-27 grade was significantly associated with worse PFS, CSS, and OS. In multivariate Cox regression and competing risk analyses, we found that ACE-27 grade, tumor stage, and tumor grade were independent prognosticators of OS, CSS, and PFS. We combined these three significant factors to construct a prognostic model for predicting clinical outcomes. A receiver operating characteristic curve revealed that our prognostic model had high predictive performance. The Harrel’s concordance indices of this model for predicting OS, CSS, and PFS were 0.81, 0.85, and 0.85, respectively. The results suggest that the UTUC patient comorbidity burden (ACE-27) provides information on the risk for meaningful clinical outcomes of OS, CSS, and PFS.

## 1. Introduction

Upper tract urothelial carcinoma (UTUC) is a rare but potentially lethal malignancy that accounts for about 5% of urothelial cancers and 10% of all renal tumors [1,2,3]. Radical nephroureterectomy (RNU) with the bladder cuff excision is the standard procedure for UTUC, despite the tumor location in the upper urinary tract [2,3,4]. Predictors of survival after RNU include histologic grade, lymphovascular invasion, pathological TNM stage, concomitant carcinoma in situ, tumor multifocality, and architecture [2,3,4]. Recent studies have focused on the genetic features of UTUC using next-generation sequencing and identified different molecular classifications with distinct clinical behaviors [5,6,7]. However, despite the refinement of the surgical and medical management of UTUC, patient outcomes have not changed significantly over the past few decades [8,9]. 

In addition, the world’s population is aging, and UTUC is associated with older age, with a peak incidence in people in their 70s and 80s, and with a high prevalence of comorbidities [2,3,8,9]. Comorbidity is defined as any coexisting condition or disease that can affect the diagnosis, treatment, and prognosis of an index disease under study [10,11]. It is an important independent prognostic factor in patients with cancer [12,13,14]. Correlations between the severity of comorbidities and clinical outcomes have been found among patients with colorectal cancer [15,16], breast cancer [16,17], prostate cancer [18,19], and bladder cancer [20].

However, the effect of comorbidities on the clinical outcomes of patients with UTUC is not well-known [21]. We wanted to assess the significance of severity of comorbidities on the prognosis of patients with UTUC treated with RNU in a large cohort using the Adult Comorbidity Evaluation-27 (ACE-27), a validated comorbidity index specifically designed for patients with cancer [12].

## 2. Materials and Methods

### 2.1. Study Population

We retrospectively reviewed the charts of 495 patients who were diagnosed with UTUC and underwent RNU at our institution from January 2000 to December 2015,. Among the 495 patients, 86 were excluded from this study because 27 had lymph node invasion or distant metastasis at the time of diagnosis, 13 had bilateral synchronous UTUC, 23 had previous or concomitant urinary bladder cancers, and 23 had incomplete data. The remaining 409 patients who received RNU for primary non-metastatic UTUC were included. None of them received preoperative radiotherapy or chemotherapy. Demographic features and comorbidity information were obtained retrospectively based on prospectively documented medical records and structured admission sheets. This study was approved by an institutional review board (KMUHIRB-E(I)-20190107).

### 2.2. Adult Comorbidity Evaluation-27

ACE-27 is a validated 27-item comorbidity index for adult oncology patients [12]. Comorbidity was defined as previous or coexisting medical diseases at the time of UTUC diagnosis. In ACE-27, specific diseases are graded into one of three levels of organ decompensation: grade 3 (severe decompensation), grade 2 (moderate decompensation), and grade 1 (mild decompensation). An example of scoring in the case of the cardiovascular system is that an old myocardial infarction (MI) (by electrocardiography only, age undetermined), a prior history of MI more than six months, or within six months would be scored as grade 1, grade 2, and grade 3 comorbidities, respectively. An overall comorbidity score (none, mild, moderate, or severe) is designated according to the highest graded disease. In patients who have two or more moderate grades in the different organ systems or disease groups, the overall ACE-27 score is assigned as severe. The tool can be found at https://www.jpiccirillo.com/ace27/ (accessed on 1 June 2019).

### 2.3. Postoperative Follow-Up

Clinicopathological data were retrospectively recorded. Tumor grade was determined based on the 2004 WHO classification. TNM staging was defined according to the 2010 American Joint Committee on Cancer classification. After RNU, postoperative follow-up consisted of detailed medical history, physical examination, urine cytology, urinalysis, and cystoscopy every three months for the first two years, every six months for the next two years, and annually thereafter. Imaging studies such as abdominal ultrasound and computed tomography or magnetic resonance imaging of the abdomen were conducted annually during follow-up or when clinically indicated. We defined UTUC progression as any recurrence in the regional lymph nodes, operative field, or distant metastasis. Medical chart reviews confirmed the causes of death.

### 2.4. Statistical Analyses

We used Pearson’s chi-square test to study the association of ACE-27 scores and clinicopathological features. Person-month is an estimate of the actual time at risk that all patients contributed to a study. It is a measurement of observation time per patient. We determine for each patient the actual time at risk from the date of RNU until cancer progression, patient death or lost follow-up, or the end of our study. Univariate and multivariate analyses were performed to assess the prognostic factors of progression-free survival (PFS), cancer-specific survival (CSS), and overall survival (OS) using a Cox proportional hazards regression model and the Kaplan-Meier method. The adjusted hazard ratio (HR) is the adjustment of all or selected covariates in the multivariate Cox regression model. As other causes of death are competing risks for PFS and CSS, we also performed a competing risk analysis using the cumulative incidence function and the Fine-Gray subdistribution hazard model [22,23]. A prognostic model was established based on the independent risk factors. The discriminative ability and predictive accuracy of the model were assessed using the area under (AUC) the receiver operating characteristic curve (ROC) and Harrel’s concordance index (C-index). Statistical analyses were performed using SAS, version 9.4 (SAS Institute Inc., Cary, NC, USA) and statistical significance was set at *p* < 0.05.

## 3. Results

### 3.1. Clinicopathological Characteristics of Patients

We included 409 patients, 183 males and 286 females, in the current study, with a median age of 69.5 years. Table 1 shows the demography of the study participants. Baseline ACE-27 comorbidity grades were as follows: severe, 43 (10.5%) patients; moderate, 139 (34.8%); mild, 174 (42.5%); none, 53 (13.0%). The distribution of pathological tumor stage was 175 (42.8%), 112 (27.4%), 106 (25.9%), and 16 (3.9%) for pTa/Tis/T1, T2, T3, and T4, respectively. Approximately 80% of the patients were diagnosed with a high-grade disorder. Regarding the primary tumor location, 213 (52.1%) were in the renal pelvis and 196 (47.6%) were located in the ureter. Approximately 10% of the patients had multiple tumors at diagnosis. High ACE-27 grade significantly correlated with old age, multiple tumors, and high tumor stage. This section may be divided by subheadings. It should provide a concise and precise description of the experimental results, their interpretation, as well as the experimental conclusions that can be drawn.

### 3.2. ACE-27 Comorbidity and Survival

**Figure 1:**
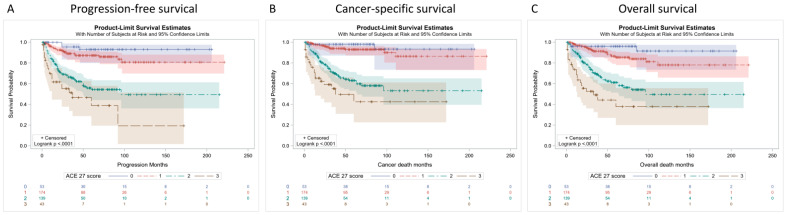
Kaplan–Meier survival curves of progression-free survival (**A**); cancer-specific survival (**B**) and overall survival (**C**) according to the ACE-27 score: 0, none; 1, mild; 2, moderate; 3, severe.

Overall, 100 (24.4%) patients experienced tumor recurrence or developed metastases during follow-up; three patients, classified as ACE-27 none, 22 as ACE-27 mild, 54 as ACE-27 moderate, and 21 as ACE-27 severe, had five-year PFS rates of 93.0%, 87.3%, 55.9%, and 38.8%, respectively (Figure 1A, *p* < 0.001). The median follow-up period was 45 months (range 1–221 months). A total of 80 patients (19.6%) died of UTUC during follow-up, of which 2, 12, 47, and 19 were patients classified as ACE-27 none, mild, moderate, and severe, respectively. The five-year estimated CSS was significantly different for the ACE-27 scores (98.1% for ACE-27 none, 93.0% for ACE-27 mild, 63.1% for ACE-27 moderate, and 42.6% for ACE-27 severe; *p* < 0.001) (Figure 1B). In terms of OS, 97 of 409 patients (23.7%) died of all causes during follow-up: three were ACE-27 none, 22 were ACE-27 mild, 50 were ACE-27 moderate, and 22 were ACE-27 severe patients. The Kaplan-Meier method estimated that the 5-year OS rates were 96.2%, 85.5%, 61.2%, and 38.0% for each group, respectively (Figure 1C, *p* < 0.001).

### 3.3. Cox Regression Analysis of Clinical Outcomes

The associations between clinicopathological characteristics and survival outcomes are shown in Table 2, Table 3 and Table 4. In univariate analysis, PFS, CSS, and OS were worse with multiple tumors, higher primary tumor stage, higher tumor grade, and increasing ACE-27 score. After adjusting for these factors, a higher ACE-27 score remained a significant prognosticator of higher disease progression and worse patient survival. Patients with moderate comorbidity had a 6.59-fold, 8.59-fold, and 6.63-fold increased risk of cancer progression, cancer-related death, and overall death, respectively, compared to patients with no comorbidity. In addition, patients with severe comorbidity had a 12.41-fold, 15.94-fold, and 13.18-fold increased risk of UTUC progression, UTUC-related death, and all-cause mortality, respectively, compared to patients with comorbidity scores of none. Primary tumor stage and grade remained independent predictors of PFS, CSS, and OS.

### 3.4. Competing Risk Analysis of Clinical Outcomes

**Figure 2:**
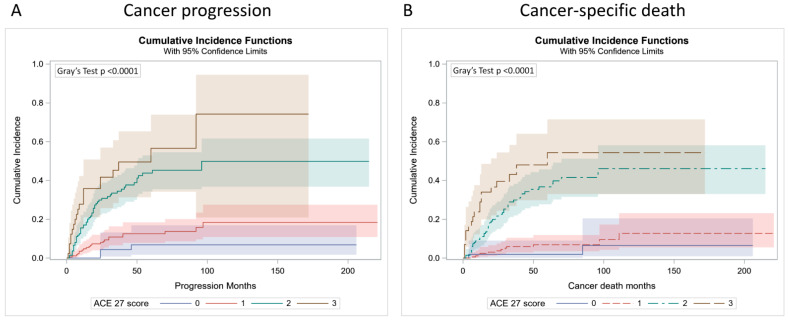
Cumulative incidence function for cancer progression (**A**) and cancer-specific death (**B**) according to the ACE-27 score: 0, none; 1, mild; 2, moderate; 3, severe.

During follow-up, patients might die from other causes before the occurrence of UTUC progression and UTUC-specific death. To accurately evaluate the prognostic value of the ACE-27 score in UTUC, a competing risk model was applied. Multivariate analysis showed that increasing ACE-27 score, higher primary tumor stage, and higher tumor grade were all significant prognostic factors for PFS (Table 5) and CSS (Table 6). Finally, the cumulative incidence function was calculated to determine the probability of cancer progression and cancer-specific death. The results demonstrated that patients with high ACE-27 scores had significantly higher cancer progression rates and cancer-specific death rates than those with low ACE-27 scores (both *p* < 0.0001, Figure 2).

### 3.5. Establish a Prognostic Model

**Figure 3:**
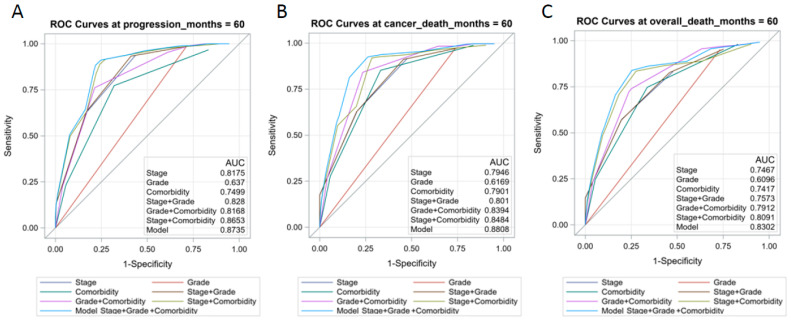
Receiver operator characteristic (ROC) analysis of seven models for predicting progression-free survival (**A**); cancer-specific survival (**B**) and overall survival (**C**) at five years.

**Figure 4:**
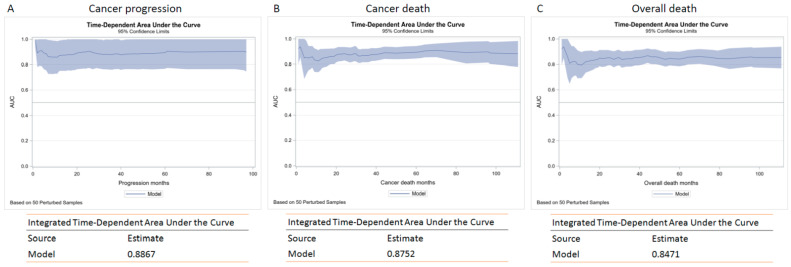
The integrated time-dependent AUC for progression-free survival (**A**); cancer-specific survival (**B**) and overall survival (**C**).

For clinical application, we built a prognostic model by combining the tumor grade, ACE-27 comorbidity index, tumor stage, and GCS model. Furthermore, we used Harrell’s concordance index and time-dependent ROC curve to test the accuracy and discrimination of the GCS model. The results demonstrated that the GCS model had the best C-index, and the value was 0.85 for cancer progression, 0.85 for cancer-related death, and 0.81 for overall death, suggesting a good discriminative capability of this model. The time-dependent ROC curve also indicated that the AUC of the GCS model was higher at one, three, and five years, compared with other models (Figure 3 and Figure S1). The integrated time-dependent AUCs, which averaged all available AUC statistics over time, were 0.887, 0.875, and 0.847 for PFS, CSS, and OS, respectively (Figure 4).

## 4. Discussion

Upper tract urothelial carcinoma is an aggressive and uncommon malignancy. Even though radical surgery is regarded as the most effective treatment for UTUC, the tumor has already advanced or metastasized at the time of diagnosis in some patients [2,3]. Recurrence is quite common among patients with UTUC after surgery, resulting in patient death [2,3]. Compared to other solid cancers, knowledge of the biological characteristics of UTUC is still limited. Some clinical, pathological, and molecular features have been evaluated for their relationship with oncological outcomes [4,5,6,7]. However, the association between comorbidity and patient prognosis in UTUC is not well known. Therefore, a large retrospective cohort study was conducted to assess the significance of comorbidities on the PFS, CSS, and OS of patients with UTUC. We used the ACE-27 to determine the severity of comorbidities [12]. Our results found that increasing the ACE-27 score was significantly associated with decreased progression-free, cancer-specific, and overall survival rates. The ACE-27 score was an independent prognostic factor after adjusting for tumor stage and grade in the multivariate analysis. Furthermore, we demonstrated that incorporating tumor grade, comorbidity, and tumor stage to develop a prognostic model had the best predictive accuracy for PFS, CSS, and OS.

Patients with cancer usually have comorbidities that may influence decision-making, therapeutic protocols and clinical outcomes [10,11]. The severity of comorbidity extremely affects patient prognosis in a dose-dependent manner in many cancer types, independent of the cancer stage [12,13,14]. In a retrospective study, Garg et al. evaluated the prevalence of 48 chronic conditions in 390,179 patients [24]. They found that patients with bladder or kidney cancer had a higher chronic comorbid condition than the general population. However, there is little information available regarding the impact of comorbidities on UTUC after RNU. This is the first study to elucidate the prognostic significance of ACE-27 in UTUC. The ACE-27 combines the Charlson comorbidity index (CCI), Kaplan-Feinstein index and previously researched comorbidities. It is a 27-item chart-based comorbidity tool developed by Piccirillo et al. for adult cancer patients and classifies comorbid conditions into three grades based on severity. [12] ACE-27 is the favorite comorbidity instrument for our study, because it was developed and validated in an observational prospective study of about 18,000 patients with cancer that confirmed a significant association with overall survival independent of cancer stage [12].

Patients with UC are old and medically complex [2,24,25]. Several researchers have found the correlation between the severity of comorbidity and patient prognosis after radical cystectomy. Fairey et al. enrolled 468 patients from a multi-institutional cystectomy database and used the ACE-27 to evaluate the significance of comorbidity on clinical outcomes [26]. They found that high comorbidity grade independently correlated with bladder CSS and OS after adjusting for tumor stage, positive surgical margin, lymphovascular invasion, and lymph node metastasis. Megwalu et al. included 675 patients with bladder cancer and determined the value of ACE-27 index on OS [27]. In multivariate analysis, they demonstrated that increasing comorbidity was associated with worse OS in patients with non-muscle invasive bladder cancer or those who received radical surgery.

The prognostic impact of other comorbidity assessment tools has also been evaluated for bladder cancer. Koppie et al. evaluated the comorbidity of bladder cancer progression and OS after radical cystectomy by using the age-adjusted CCI (ACCI) [28]. The results showed that a high ACCI score was associated with high extravesical disease, low lymph node dissection, removal of fewer lymph nodes, and less postoperative chemotherapy. A higher ACCI significantly predicted a lower overall survival rate. Moreover, Eisenberg et al. incorporated the CCI, Eastern Cooperative Oncology Group (ECOG) performance status (PS), and clinicopathological features to establish the Survival Prediction After Radical Cystectomy (SPARC) score to estimate CSS in patients who underwent radical cystectomy [29]. In the multivariate analysis, ECOG PS and CCI significantly correlated with bladder CSS. They used cumulative scores to stratify patients into risk groups with significantly different five-year CSS rates. The concordance index of the SPARC model was 0.75. These findings suggest the importance of comorbidities in bladder cancer survival.

However, few studies have evaluated the impact of comorbidity on patient survival in UTUC. Shariat and Yap et al. found that older age at the time of RNU was associated with aggressive cancer features and with an inferior survival rate [30,31]. Because our cohort was rather young, we failed to prove the significant association with age and oncological outcomes. Berod et al. designed a retrospective study to assess the significance of American Society of Anesthesiologists (ASA) scores on oncological outcomes [21]. Approximately 20% of patients with UTUC were classified as ASA 3. During follow-up, 15.9% of patients died of UTUC, 37.5% experienced recurrence, and 19.7% developed metastases. They found that ASA scores significantly correlated with CSS in multivariate analysis but not with recurrence-free survival (RFS) and metastasis-free survival. They suggested incorporating ASA status into risk prediction models to improve their accuracy. Yuan et al. also evaluated the prognostic impact of ASA scores in UTUC after RNU. They demonstrated that higher ASA scores were independently associated with poor metastasis-free survival, CSS, and OS [32]. Martinez-Salamanca et al. estimated the prognostic roles of ECOG-PS, including oncological outcomes and perioperative mortality, in a large international multicenter cohort [33]. Of the UTUC patients with ECOG-PS ≥ 1, 36% had lower five-year CSS, OS, and RFS compared with patients with ECOG-PS = 0. ECOG-PS status was not a significant prognostic factor of either CSS or RFS, but independently predicted OS in the multivariate analysis model. Bagrodia et al. enrolled 835 UTUC patients who underwent either RNU or partial ureterectomy to assess the oncological outcomes [34]. They found that ECOG-PS, tumor stage, lymph node status, and tumor necrosis were significantly associated with CSS in the multivariate analysis. Aziz et al. enrolled 242 UTUC patients from three German academic centers to investigate the predictive capacity of ECOG-PS, CCI, ACCI, and ASA score [35]. They found different comorbidity and performance indices have different prognostic values. ECOG-PS > 1 and ACCI > 5 were associated with worse OS, CSS, and RFS, and ACCI > 5 and ASA-score ≥ 3 with high cancer-independent mortality in Kaplan-Meier analyses. In multivariable analysis, ECOG-PS > 1 and ASA-score ≥ 3 were independent predictors for CSS and cancer-independent mortality, respectively. 

Comorbidity assessment approaches vary in their advantages and disadvantages [36,37]. Sarfati did a comprehensive review for 21 comorbidity indices in the cancer population [36]. For comparison of these indices, content/face validity, concurrent validity, predictive validity, reliability, and feasibility were evaluated. He concluded that no gold standard measure of comorbidity existed. Among these indices, ACE27, National Cancer Institute Index, Elixhauser approach, and CCI scored moderately well on the above mentioned validity criteria. ACE-27 was specifically designed and validated in predicting outcomes in cancer patients [12,37,38]. Sarfati commented that ACE-27 was developed and extensively used among cancer patient populations. It included most relevant comorbidities and made reasonable scoring assumptions. The concurrent validity, predictive validity, and reliability of ACE-27 were supported by strong evidence. If clinical data are available, ACE-27 would be a good option [36]. To the best of our knowledge, this is the first study to incorporate ACE-27 into the prognostic model in UTUC. Our results showed that ACE-27 status was an independent prognostic factor for clinical outcomes after adjusting for tumor characteristics. UTUC patients with severe ACE-27 had the worst five-year OS, CSS, and PFS. Harrell’s concordance index of OS, CSS, and PFS was significantly improved in our GCS model. Compared with the tumor stage and grade, the integration of ACE-27 could enhance separate and discriminatory abilities.

This study had several limitations. First, the retrospective assessment of ACE-27 comorbidity scores could have led to a bias, although the scores were reviewed by two urologists independently. Second, patients enrolled in a high-volume tertiary care center may have advanced tumor stage and high comorbidity. Third, various urologists have performed open or laparoscopic techniques to treat patients over a long period of time. However, both surgical methods have similar oncological outcomes. Fourth, for a homogeneous study cohort, some selection bias may be present because we excluded patients with metastatic disease, bilateral synchronous UTUC, previous or concomitant urinary bladder cancers, and incomplete data. Despite these limitations, our study had some strengths, for instance a centralized pathological review, a large UTUC cohort, and a standardized follow-up.

## 5. Conclusions

ACE-27 comorbidity status was significantly correlated with cancer progression, cancer-related death, and all-cause mortality. This should be considered in decision-making in UTUC patients receiving RNU. The prognostic GCS model incorporating tumor grade, ACE-27 comorbidity, and tumor stage showed the best predictive capacity. Further research is needed to validate our model in well-designed, prospective, multicenter studies.

## Figures and Tables

**Table 1:** The association of ACE-27 and clinicopathologic characteristics.

	Total Patients (*n* = 409)	ACE-27, None (*n* = 53)	ACE-27, Mild (*n* = 174)	ACE-27, Moderate (*n* = 139)	ACE-27, Severe (*n* = 43)	*p* Value
Age, (years)						
≤65,	182 (44.5)	33 (62.3)	72 (41.4)	62 (44.6)	15 (34.9)	
>65	227 (55.5)	20 (37.7)	102 (58.6)	77 (55.4)	28 (65.1)	0.0284 *
Sex						
Female	226 (55.3)	30 (56.6)	88 (50.6)	84 (60.4)	24 (55.8)	
Male	183 (44.7)	23 (43.4)	86 (49.4)	55 (39.6)	19 (44.2)	0.3776
Side						
Right	186 (45.5)	23 (43.4)	82 (47.1)	58 (41.7)	23 (53.5)	
Left	223 (54.5)	30 (56.6)	92 (52.9)	81 (58.3)	20 (46.5)	0.5349
Tumor location						
Renal pelvis	213 (52.1)	28 (52.8)	91 (52.3)	74 (53.2)	20 (46.5)	
Ureter	196 (47.9)	25 (47.2)	83 (47.7)	65 (46.8)	23 (53.5)	0.8909
Mutifocality						
No	323 (79.0)	49 (92.5)	140 (80.5)	99 (71.2)	35 (81.4)	
Yes	86 (21.0)	4 (7.5)	34 (19.5)	40 (28.8)	8 (18.6)	0.0106 *
Type op RNU						
Open	294 (71.9)	40 (75.5)	120 (69.0)	103 (74.1)	31 (72.1)	
Laparoscopy	115 (28.1)	13 (24.5)	54 (31.0)	36 (25.9)	12 (27.9)	0.7033
Tumor stage (pT)						
1	175 (42.8)	31 (58.5)	82 (47.1)	50 (36.0)	12 (27.9)	
2	112 (27.4)	10 (18.9)	49 (28.2)	39 (28.1)	14 (32.6)	
3	106 (25.9)	12 (22.6)	42 (24.1)	42 (30.2)	10 (23.3)	
4	16 (3.9)	0 (0.0)	1 (0.6)	8 (5.8)	7 (16.3)	<0.0001 *
Tumor grade						
Low	86 (21.0)	18 (34.0)	36 (20.7)	26 (18.7)	6 (14.0)	
High	323 (79.0)	35 (66.0)	138 (79.3)	113 (81.3)	37 (86.0)	0.0688

Values are percentage (%). Chi-square is a statistical test used to examine the differences between categorical variables. * *p* < 0.05.

**Table 2:** Cox Regression Analysis of Prognostic Factors for Progression-free Survival.

	Event/Total Patients, %	Person-Months	Incidence Rate (95% CI) per 1000 Person-Months	Crude HR (95% CI)	*p* Value	Adjusted HR (95% CI)	*p* Value	Adjusted (Selection) HR (95% CI)	*p* Value
Age, (years)									
≤65,	49/182, 26.92	10,553	4.64 (4.56–4.73)	1.00		1.00			
>65	51/227, 22.47	11,079	4.60 (4.52–4.69)	0.88 (0.59–1.30)	0.5184	0.72 (0.48–1.08)	0.1081		
Sex									
Female	59/226, 26.11	11,575	5.10 (5.01–5.19)	1.00		1.00			
Male	41/183, 22.40	10,057	4.08 (4.00–4.16)	0.84 (0.56–1.25)	0.3825	0.74 (0.49–1.11)	0.1470		
Side									
Right	49/186, 26.34	10,066	4.87 (4.77–4.96)	1.00		1.00			
Left	51/223, 22.87	11,566	4.41 (4.33–4.49)	0.89 (0.60–1.32)	0.5776	0.86 (0.57–1.29)	0.4532		
Tumor location									
Renal pelvis	47/213, 22.07	11,801	3.98 (3.91–4.06)	1.00		1.00			
Ureter	53/196, 27.04	9831	5.39 (5.29–5.50)	1.29 (0.87–1.91)	0.2009	1.57 (1.01–2.43)	0.0440		
Mutifocality									
No	72/323, 22.29	17,010	4.23 (4.17–4.30)	1.00		1.00		1.00	
Yes	28/86, 32.56	4622	6.06 (5.89–6.24)	1.66 (1.07–2.57)	0.0232 *	1.18 (0.71–1.95)	0.5217	0.99 (0.62–1.58)	0.9733
Type op RNU									
Open	68/294, 23.13	15,990	4.25 (4.19–4.32)	1.00		1.00			
Laparoscopy	32/115, 27.83	5642	5.67 (5.53–5.82)	1.20 (0.79–1.82)	0.4023	1.20 (0.77–1.84)	0.4205		
Tumor stage (pT)									
1	8/175, 4.57	11,727	0.68 (0.67–0.69)	1.00		1.00		1.00	
2	30/112, 26.79	5791	5.18 (5.05–5.32)	6.87 (3.15–14.99)	<0.0001 *	3.74 (1.66–8.46)	0.0015 *	3.73 (1.68–8.32)	0.0013 *
3	49/106, 46.23	3963	12.36 (11.99–12.76)	15.01 (7.09–31.78)	<0.0001 *	8.38 (3.81–18.41)	<0.0001 *	7.60 (3.50–16.49)	<0.0001 *
4	13/16, 81.25	151	86.09 (73.4–100.98)	72.61 (29.36–179.56)	<0.0001 *	21.69 (8.30–56.72)	<0.0001 *	21.18 (8.22–54.62)	<0.0001 *
Tumor grade									
Low	1/86, 1.16	6409	0.16 (0.15–0.16)	1.00		1.00		1.00	
High	99/323, 30.65	15,223	6.50 (6.40–6.61)	33.42 (4.66–239.73)	0.0005 *	8.91 (1.15–68.86)	0.0360 *	9.61 (1.26–73.26)	0.0289 *
ACE-27 Comorbidity									
None, 0	3/53, 5.66	4255	0.71 (0.68–0.73)	1.00		1.00		1.00	
Mild, 1	22/174, 12.64	10,266	2.14 (2.10–2.18)	2.54 (0.76–8.48)	0.1308	2.28 (0.68–7.68)	0.1832	2.25 (0.67–7.54)	0.1904
Moderate, 2	54/139, 38.85	5929	9.11 (8.88–9.34)	9.56 (2.98–30.66)	0.0001 *	6.59 (2.02–21.49)	0.0018 *	6.97 (2.14–22.69)	0.0013 *
Severe, 3	21/43, 48.84	1182	17.77 (16.78–18.81)	17.11 (5.07–57.68)	<0.0001 *	12.41 (3.59–42.84)	<0.0001 *	11.58 (3.40–39.47)	<0.0001 *

Hazard ratios (HR) with 95% confidence interval (CI) was calculated by using Cox proportional hazards regression model. * *p* < 0.05.

**Table 3:** Cox Regression Analysis of Prognostic Factors for Cancer-specific Survival.

	Event/Total Patients, %	Person-Months	Incidence Rate (95% CI) per 1000 Person-Months	Crude HR (95% CI)	*p* Value	Adjusted HR (95% CI)	*p* Value	Adjusted (Selection) HR (95% CI)	*p* Value
Age, (years)									
≤65,	42/182, 23.08	11,320	3.71 (3.64–3.78)	1.00		1.00			
>65	38/227, 16.74	11,652	3.26 (3.20–3.32)	0.79 (0.51–1.22)	0.2862	0.62 (0.39–0.98)	0.0413		
Sex									
Female	45/226, 19.91	12,472	3.61 (3.55–3.67)	1.00		1.00			
Male	35/183, 19.13	10,500	3.33 (3.27–3.40)	0.94 (0.61–1.47)	0.7985	0.91 (0.58–1.45)	0.6972		
Side									
Right	38/186, 20.43	10,532	3.61 (3.54–3.68)	1.00		1.00			
Left	42/223, 18.83	12,440	3.38 (3.32–3.44)	0.93 (0.6–1.45)	0.7592	0.87 (0.55–1.36)	0.5342		
Tumor location									
Renal pelvis	37/213, 17.37	12,328	3.00 (2.95–3.05)	1.00		1.00			
Ureter	43/196, 21.94	10,644	4.04 (3.96–4.12)	1.32 (0.85–2.04)	0.2214	1.60 (0.98–2.60)	0.0597		
Mutifocality									
No	55/323, 17.03	18,086	3.04 (3.00–3.09)	1.00		1.00		1.00	
Yes	25/86, 29.07	4886	5.12 (4.98–5.26)	1.87 (1.16–3.00)	0.0095 *	1.43 (0.83–2.48)	0.1975	1.14 (0.69–1.89)	0.6011
Type op RNU									
Open	59/294, 20.07	16,849	3.50 (3.45–3.55)	1.00		1.00			
Laparoscopy	21/115, 18.26	6123	3.43 (3.34–3.52)	0.89 (0.54–1.46)	0.6435	0.93 (0.56–1.54)	0.7781		
Tumor stage (pT)									
1	9/175, 5.14	12,111	0.74 (0.73–0.76)	1.00		1.00		1.00	
2	22/112, 19.64	5956	3.69 (3.60–3.79)	4.51 (2.07–9.80)	0.0001 *	2.59 (1.13–5.95)	0.0247 *	2.70 (1.20–6.10)	0.01670
3	37/106, 34.91	4733	7.82 (7.6–8.04)	9.16 (4.41–19.04)	<0.0001 *	4.65 (2.10–10.29)	0.0002 *	4.72 (2.16–10.27)	<0.0001 *
4	12/16, 75.00	172	69.77 (60.08–81.01)	58.43 (23.73–143.88)	<0.0001 *	15.43 (5.85–40.70)	<0.0001 *	16.00 (6.13–41.8)	<0.0001 *
Tumor grade									
Low	2/86, 2.33	6483	0.31 (0.30–0.32)	1.00		1.00			
High	78/323, 24.15	16,489	4.73 (4.66–4.80)	12.78 (3.14–52.06)	0.0004 *	4.60 (1.02–20.78)	0.0475 *	4.45 (1.00–19.88)	0.0504
ACE-27 Comorbidity									
None, 0	2/53, 3.77	4342	0.46 (0.45–0.47)	1.00		1.00		1.00	
Mild, 1	12/174, 6.9	10,781	1.11 (1.09–1.13)	2.07 (0.46–9.24)	0.342	1.86 (0.41–8.37)	0.4178	1.81 (0.40–8.12)	0.4386
Moderate, 2	47/139, 33.81	6506	7.22 (7.05–7.40)	12.26 (2.97–50.61)	0.0005 *	8.59 (2.04–36.11)	0.0033 *	8.82 (2.10–36.94)	0.0029 *
Severe, 3	19/43, 44.19	1343	14.15 (13.41–14.92)	22.4 (5.19–96.58)	<0.0001 *	15.94 (3.63–70.03)	0.0002 *	13.90 (3.19–60.48)	0.0005 *

Hazard ratios (HR) with 95% confidence interval (CI) was calculated by using Cox proportional hazards regression model. * *p* < 0.05.

**Table 4:** Cox Regression Analysis of Prognostic Factors for Overall Survival.

	Event/Total Patients, %	Person-Months	Incidence Rate (95% CI) per 1000 Person-Months	Crude HR (95% CI)	*p* Value	Adjusted HR (95% CI)	*p* Value	Adjusted (Selection) HR (95% CI)	*p* Value
Age, (years)									
≤65,	46/182, 25.27	11,320	4.06 (3.99–4.14)	1.00		1.00			
>65	51/227, 22.47	11,652	4.38 (4.30–4.46)	0.97 (0.65–1.45)	0.8858	0.78 (0.52–1.18)	0.2459		
Sex									
Female	55/226, 24.34	12,472	4.41 (4.33–4.49)	1.00		1.00			
Male	42/183, 22.95	10,500	4.00 (3.92–4.08)	0.93 (0.62–1.39)	0.7173	0.87 (0.58–1.32)	0.5247		
Side									
Right	46/186, 24.73	10,532	4.37 (4.29–4.45)	1.00		1.00			
Left	51/223, 22.87	12,440	4.10 (4.03–4.17)	0.94 (0.63–1.41)	0.7739	0.88 (0.58–1.32)	0.5217		
Tumor location									
Renal pelvis	43/213, 20.19	12,328	3.49 (3.43–3.55)	1.00		1.00			
Ureter	54/196, 27.55	10,644	5.07 (4.98–5.17)	1.42 (0.95–2.12)	0.0881	1.70 (1.10–2.64)	0.0175		
Mutifocality									
No	70/323, 21.67	18,086	3.87 (3.81–3.93)	1.00		1.00		1.00	
Yes	27/86, 31.40	4886	5.53 (5.37–5.68)	1.59 (1.02–2.49)	0.0398 *	1.31 (0.79–2.17)	0.2983	1.06 (0.67–1.69)	0.8023
Type op RNU									
Open	74/294, 25.17	16,849	4.39 (4.33–4.46)	1.00		1.00			
Laparoscopy	23/115, 20.00	6123	3.76 (3.66–3.85)	0.77 (0.48–1.24)	0.2842	0.79 (0.49–1.27)	0.3337		
Tumor stage (pT)									
1	18/175, 10.29	12,111	1.49 (1.46–1.51)	1.00		1.00		1.00	
2	25/112, 22.32	5956	4.20 (4.09–4.31)	2.58 (1.41–4.73)	0.0022 *	1.61 (0.83–3.11)	0.159	1.68 (0.88–3.2)	0.1152
3	42/106, 39.62	4733	8.87 (8.62–9.13)	5.28 (3.03–9.19)	<0.0001 *	3.14 (1.69–5.84)	0.0003 *	3.10 (1.70–5.68)	0.0002 *
4	12/16, 75.00	172	69.77 (60.08–81.01)	31.21 (14.47–67.31)	<0.0001 *	9.80 (4.24–22.64)	<0.0001 *	10.43 (4.55–23.92)	<0.0001 *
Tumor grade									
Low	5/86, 5.81	6483	0.77 (0.75–0.79)	1.00		1.00		1.00	
High	92/323, 28.48	16,489	5.58 (5.49–5.67)	6.08 (2.47–14.98)	<0.0001 *	2.97 (1.10–8.04)	0.0319 *	2.94 (1.10–7.85)	0.0316 *
ACE-27 Comorbidity									
None, 0	3/53,5.66	4342	0.69 (0.67–0.71)	1.00		1.00		1.00	
Mild, 1	22/174,12.64	10,781	2.04 (2–2.08)	2.56 (0.77–8.56)	0.1268	2.35 (0.70–7.89)	0.1675	2.34 (0.70–7.85)	0.1684
Moderate, 2	50/139,35.97	6506	7.69 (7.5–7.87)	8.93 (2.78–28.69)	0.0002*	6.63 (2.03–21.66)	0.0018 *	6.91 (2.12–22.5)	0.0013 *
Severe, 3	22/43,51.16	1343	16.38 (15.53–17.28)	18.08 (5.39–60.66)	<0.0001 *	13.18 (3.86–45.00)	<0.0001 *	12.16 (3.59–41.2)	<0.0001 *

Hazard ratios (HR) with 95% confidence interval (CI) was calculated by using Cox proportional hazards regression model. * *p* < 0.05.

**Table 5:** Competing Risk Analysis for Progression-free Survival.

	Crude SHR (95% CI)	*p* Value	Adjusted SHR (95% CI)	*p* Value	Adjusted (Selection) SHR (95% CI)	*p* Value
Age, (years)						
≤65,	1.00		1.00			
>65	0.85 (0.57–1.25)	0.4054	0.68 (0.45–1.05)	0.0821		
Sex						
Female	1.00		1.00			
Male	0.85 (0.57–1.27)	0.4381	0.77 (0.51–1.17)	0.2167		
Side						
Right	1.00		1.00			
Left	0.92 (0.62–1.36)	0.6739	0.89 (0.58–1.35)	0.5775		
Tumor location						
Renal pelvis	1.00		1.00			
Ureter	1.25 (0.84–1.85)	0.2692	1.46 (0.95–2.23)	0.0814		
Mutifocality						
No	1.00		1.00		1.00	
Yes	1.71 (1.1–2.67)	0.0166 *	1.21 (0.69–2.12)	0.5028	1.03 (0.62–1.7)	0.9124
Type op RNU						
Open	1.00		1.00			
Laparoscopy	1.23 (0.81–1.88)	0.3250	1.31 (0.81–2.1)	0.2698		
Tumor stage (pT)						
1	1.00		1.00		1.00	
2	6.59 (3.05–14.28)	<0.0001 *	3.60 (1.61–8.02)	0.0018 *	3.54 (1.62–7.73)	0.0015 *
3	14.93 (7.14–31.26)	<0.0001 *	8.30 (3.83–18.00)	<0.0001 *	7.64 (3.56–16.38)	<0.0001 *
4	73.77 (31.47–172.94)	<0.0001 *	22.75 (9.20–56.26)	<0.0001 *	21.83 (9.07–52.56)	<0.0001 *
3 + 4 vs. 1 + 2	11.31 (5.56–23.03)	<0.0001 *	6.34 (3.05–13.19)	<0.0001 *	5.84 (2.83–12.05)	<0.0001 *
Tumor grade						
Low	1.00		1.00		1.00	
High	32.56 (4.57–232.22)	0.0005 *	8.86 (1.19–66.18)	0.0334 *	9.39 (1.28–68.78)	0.0275 *
ACE-27 Comorbidity						
None, 0	1.00		1.00		1.00	
Mild, 1	2.51 (0.76–8.29)	0.1319	2.23 (0.69–7.16)	0.1777	2.17 (0.69–6.87)	0.1874
Moderate, 2	9.52 (3.01–30.13)	0.0001 *	6.43 (2.06–20.07)	0.0013 *	6.69 (2.17–20.65)	0.0009 *
Severe, 3	14.93 (4.46–50.02)	<0.0001 *	10.82 (3.28–35.68)	<0.0001 *	10.12 (3.15–32.50)	0.0001 *
2 + 3 vs. 0 + 1	4.95 (3.16–7.77)	<0.0001 *	4.44 (2.79–7.07)	<0.0001 *	4.47 (2.81–7.10)	<0.0001 *

Abbreviations: SHR, subdistribution hazard ratio; 95% CI, 95% confidence intervals. * *p* < 0.05.

**Table 6:** Competing Risk Analysis for Cancer-specific Survival.

	Crude SHR (95% CI)	*p* Value	Adjusted SHR (95% CI)	*p* Value	Adjusted (Selection) SHR (95% CI)	*p* Value
Age, (years)						
≤65,	1.00		1.00			
>65	0.77 (0.50–1.20)	0.2489	0.60 (0.38–0.94)	0.0264		
Sex						
Female	1.00		1.00			
Male	0.95 (0.61–1.47)	0.8061	0.93 (0.59–1.48)	0.7645		
Side						
Right	1.00		1.00			
Left	0.94 (0.61–1.45)	0.7796	0.88 (0.56–1.38)	0.5703		
Tumor location						
Renal pelvis	1.00		1.00			
Ureter	1.29 (0.84–2.00)	0.2473	1.53 (0.97–2.40)	0.0652		
Mutifocality						
No	1.00		1.00		1.00	
Yes	1.90 (1.19–3.06)	0.0077 *	1.46 (0.83–2.54)	0.1857	1.17 (0.70–1.97)	0.5508
Type op RNU						
Open	1.00		1.00			
Laparoscopy	0.90 (0.55–1.49)	0.6921	0.97 (0.59–1.60)	0.9161		
Tumor stage (pT)						
1	1.00		1.00		1.00	
2	4.48 (2.07–9.69)	0.0001 *	2.52 (1.10–5.80)	0.0297 *	2.63 (1.18–5.89)	0.0182 *
3	9.11 (4.4–18.88)	<0.0001 *	4.65 (2.08–10.41)	0.0002 *	4.77 (2.21–10.33)	<0.0001 *
4	58.46 (24.57–139.08)	<0.0001 *	15.74 (6.37–38.86)	<0.0001 *	16.2 (6.47–40.59)	<0.0001 *
3 + 4 vs. 1 + 2	7.55 (3.79–15.05)	<0.0001 *	4.07 (1.93–8.55)	0.0002 *	4.13 (2.02–8.46)	0.0001 *
Tumor grade						
Low	1.00		1.00		1.00	
High	12.57 (3.06–51.55)	0.0004 *	4.64 (1.05–20.55)	0.0430 *	4.39 (1.02–18.93)	0.0473 *
ACE-27 Comorbidity						
None, 0	1.00		1.00		1.00	
Mild, 1	2.04 (0.46–9.08)	0.3491	1.81 (0.40–8.10)	0.4400	1.75 (0.39–7.94)	0.4657
Moderate, 2	12.19 (2.98–49.93)	0.0005 *	8.38 (2.01–34.88)	0.0035 *	8.55 (2.02–36.3)	0.0036 *
Severe, 3	20.53 (4.76–88.57)	<0.0001 *	14.81 (3.44–63.81)	0.0003 *	12.90 (2.91–57.22)	0.0008 *
2 + 3 vs. 0 + 1	7.75 (4.38–13.72)	<0.0001 *	7.05 (3.94–12.60)	<0.0001 *	6.96 (3.89–12.47)	<0.0001 *

Abbreviations: SHR, subdistribution hazard ratio; 95% CI, 95% confidence intervals. * *p* < 0.05.

## Data Availability

All data generated or analyzed during this study are included in this published article.

## Supplementary Materials

The following supporting information can be downloaded at: https://www.mdpi.com/article/10.3390/cancers14061466/s1, Figure S1: Receiver operator characteristic (ROC) analysis of seven models for predicting progression-free survival, cancer-specific survival and overall survival at 1 year (A–C) and 3 years (D–F).
