# Supplementary material for: Prognostic Value of Comorbidity for Patients with Upper Tract Urothelial Carcinoma after Radical Nephroureterectomy
Source: Cancers (Basel). 2022 Mar 12;14(6):1466. doi: 10.3390/cancers14061466 (PMC8946018; doi:10.3390/cancers14061466)
Supplement: Supplementary file 1 [file cancers-14-01466-s001.zip › cancers-1621192-supplementary.pdf]

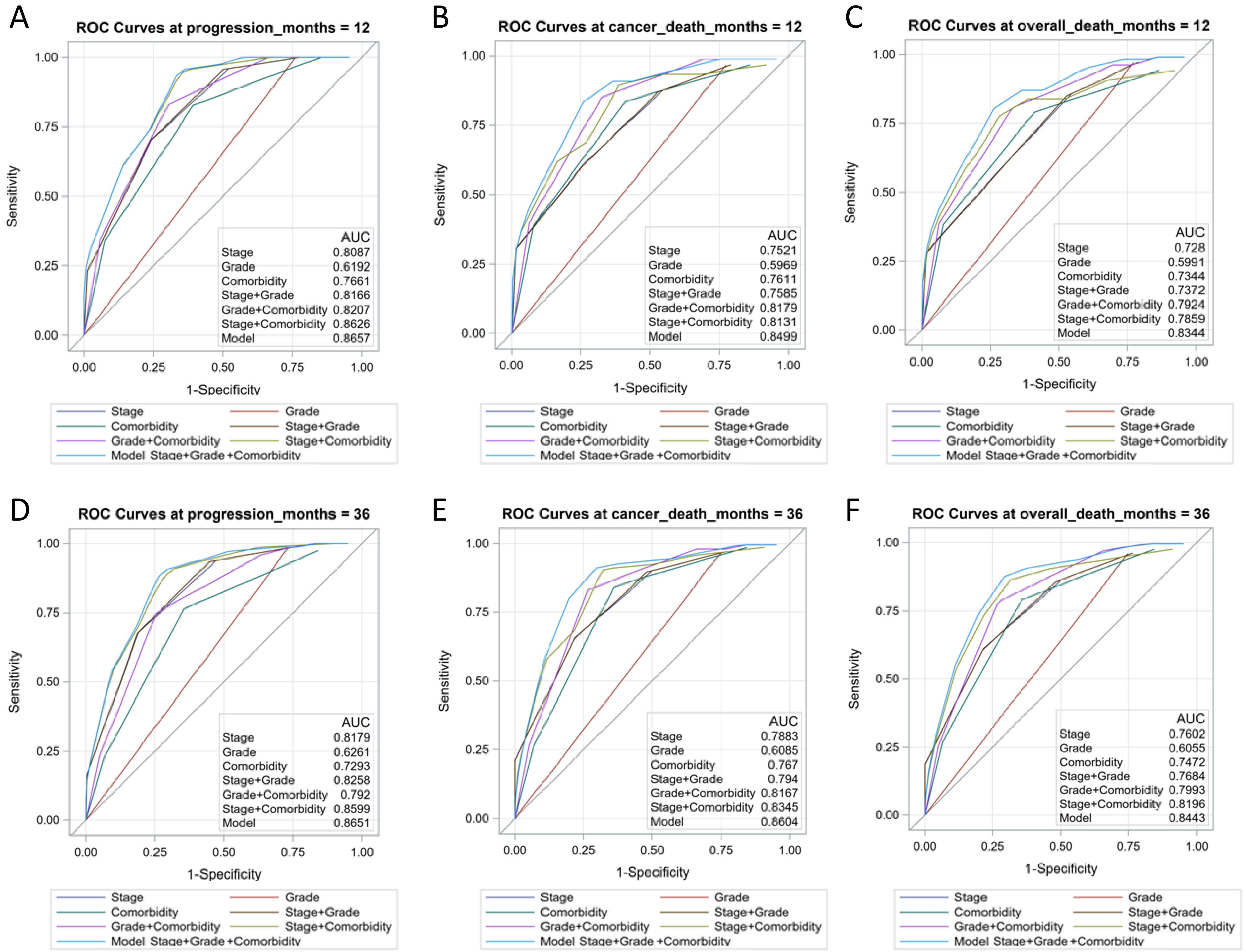

Figure S1. Receiver operator characteristic (ROC) analysis of seven models for predicting progres-sion-free survival, cancer-specific survival and overall survival at 1 year (A,B,C) and 3 years (D,E,F).
